# Mn_2_P_2_O_7_–polyaniline hybrid composite as a promising electrode material for advanced symmetric supercapacitors

**DOI:** 10.1039/d5ra04149j

**Published:** 2025-07-15

**Authors:** Eman A. Alabdullkarem, Junaid Khan

**Affiliations:** a Chemistry Department, College of Science, King Saud University Riyadh 11451 Saudi Arabia; b Department of Physics, Government Postgraduate College No.1 Abbottabad Khyber Pakhtunkhwa Pakistan junaidkhan1751996@gmail.com; c Department of Higher Education Archives and Libraries, Government of Khyber Pakhtunkhwa Pakistan; d Department of Chemical and Biological Engineering, Gachon University 1342 Seongnam-daero Seongnam 13120 Republic of Korea

## Abstract

The development of advanced electrode materials with superior electrochemical performance is imperative to meet the growing demands of modern energy storage technologies. In this study, we report a novel Mn_2_P_2_O_7_–polyaniline (PMP) nanohybrid synthesized *via in situ* oxidative polymerization of aniline onto hydrothermally prepared Mn_2_P_2_O_7_ (MP) nanoclusters. The resulting materials were thoroughly characterized using structural, morphological, chemical, and elemental analysis techniques. The electrochemical behavior of the PMP nanohybrid was systematically investigated and compared with the pristine MP electrode. The PMP electrode exhibited substantial enhancement in performance, achieving a 1.78-fold increase in specific capacity, a 44% improvement in rate capability at 20 A g^−1^, and excellent cycling stability. Notably, a symmetric supercapacitor device assembled using PMP delivered a high energy density of 79.1 Wh kg^−1^ at a power density of 749.3 W kg^−1^ while retaining 90.5% of its initial capacity after 10 000 GCD cycles, underscoring its outstanding long-term durability. These findings demonstrate that the PMP nanohybrid offers a promising strategy for engineering high-performance, stable, and sustainable supercapacitor devices, paving the way for practical applications in next-generation energy storage systems.

## Abstract 2

The development of advanced electrode materials with superior electrochemical performance is imperative to meet the growing demands of modern energy storage technologies.

## Graphical abstract

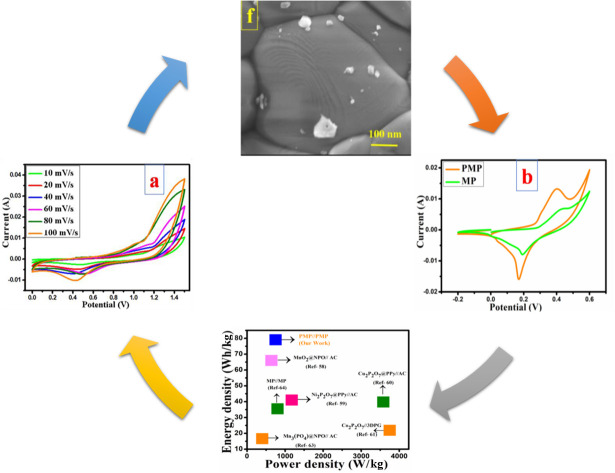

## 1. Introduction

Driven by rapid industrialization, urbanization, and technological advancement, the exponential growth in global energy consumption underscores the urgent need for efficient, sustainable, and environmentally friendly energy storage solutions. Projections indicate a potential doubling of global energy demand in the coming decades, placing immense pressure on existing infrastructure and necessitating transformative advances in energy storage technologies.^1–3^ Supercapacitors (SCs) have emerged as pivotal components in this area, uniquely bridging the gap between conventional capacitors and batteries by offering rapid charge–discharge rates, exceptional power density, and outstanding long-term cycling stability. These attributes make them indispensable for high-power applications such as electric vehicles, grid stabilization, and portable electronics.^4–6^ However, the widespread deployment of SC remains constrained by inherent trade-offs between energy density, power density, and cycling stability, largely dictated by the limitations of existing electrode materials.

Electrode materials for SCs are broadly categorized by their charge storage mechanisms. Electric double-layer capacitors (EDLCs) are exemplified by carbon-based materials. They rely on electrostatic ion adsorption but suffer from intrinsically low energy density.^7–9^ Pseudocapacitors utilize fast surface/near-surface faradaic reactions in materials like metal oxides or conductive polymers. They offer higher energy density but often face compromises in power density and cycling stability.^10–12^ Battery-type supercapacitors employ bulk diffusion-controlled faradaic processes in materials such as metal oxides, hydroxides, sulfides, or phosphates. They deliver high energy density but are typically hindered by sluggish kinetics and poor rate capability.^13–15^ Critically, recent research has focused on overcoming these limitations through the strategic design of hybrid composite electrodes, which synergistically combine materials with complementary properties. Significant advancements have been demonstrated with composites such as conductive polymer/metal oxide hybrids (*e.g.*, PANI/MnO_2_), carbon nanomaterial/metal compound composites (*e.g.*, graphene/NiCo-LDH), and ternary hybrids, leveraging conductive networks for electron transfer, redox-active components for charge storage, and tailored interfaces to enhance stability and mitigate degradation.^16–23^ Despite this progress, key challenges persist across composite systems, including the achievement of optimal interfacial contact for efficient charge transfer, ensuring uniform dispersion to prevent agglomeration, maintaining structural integrity during cycling, and developing scalable synthesis methods—all crucial for realizing high performance and reproducibility.^24–27^

Within this context, transition metal phosphates (TMPs), particularly manganese pyrophosphate (Mn_2_P_2_O_7_), represent a highly promising yet underexplored class of battery-type electrode materials. Mn_2_P_2_O_7_ offers compelling advantages: high theoretical capacitance enabled by rich Mn^2+^/Mn^3+^/Mn^4+^ redox chemistry, exceptional structural stability derived from its robust polyanionic (P_2_O_7_^4−^) framework facilitating ion diffusion, and environmental friendliness.^28–30^ However, its practical application is severely limited by its intrinsically low electronic conductivity and particle agglomeration, leading to poor rate capability, underutilization of the active material, and limited cycling stability^31–33^—a gap that necessitates innovative material design.

Hybridization with conductive polymers, specifically polyaniline (PANI), presents a powerful strategy to address these limitations. PANI is a benchmark pseudocapacitive material renowned for its high intrinsic conductivity (in doped states), multiple reversible redox states, environmental stability, ease of synthesis, and mechanical flexibility.^34–37^ Critically, recent research demonstrates that PANI-based composites (*e.g.*, with metal oxides or sulfides) exhibit markedly enhanced electrochemical properties compared to their individual constituents. This is primarily due to their improved conductivity, additional pseudocapacitance, suppression of particle aggregation, and buffering of volume changes.^38–42^ Motivated by this proven synergy and the unique advantages of Mn_2_P_2_O_7_, the Mn_2_P_2_O_7_–PANI hybrid emerges as a highly rational study object. The combination offers the potential to marry the high energy density of Mn_2_P_2_O_7_'s battery-type behavior with the high power density and conductivity of PANI's pseudocapacitance. However, there has been exceptionally scarce research studies specifically on Mn_2_P_2_O_7_–PANI composites. Furthermore, the fundamental challenges in such inorganic–organic hybrids—such as achieving strong interfacial compatibility for efficient charge transfer, ensuring uniform dispersion of the inorganic phase within the polymer matrix, avoiding structural damage during synthesis, and developing scalable fabrication routes—remain largely unaddressed for this specific system,^43–46^ presenting a clear knowledge gap and research opportunity.

To directly address this gap and the aforementioned challenges in composite design, this work introduces a novel Mn_2_P_2_O_7_–polyaniline hybrid composite (PMP) synthesized *via* a facile and scalable *in situ* oxidative polymerization strategy. This approach is designed to optimize the interfacial adhesion, ensure a homogeneous dispersion of Mn_2_P_2_O_7_ within the conductive PANI network, and preserve the component integrity. We employ a suite of characterization techniques—including field emission scanning electron microscopy (FESEM), transmission electron microscopy (TEM), X-ray diffraction (XRD), Fourier transform infrared spectroscopy (FTIR), and X-ray photoelectron spectroscopy (XPS)—to elucidate the morphology, structure, composition, and interfacial properties of PMP. Its electrochemical performance is rigorously assessed using cyclic voltammetry (CV), galvanostatic charge–discharge (GCD), and electrochemical impedance spectroscopy (EIS). Our findings demonstrate that the synergistic interaction within the PMP hybrid effectively overcomes the limitations of pristine Mn_2_P_2_O_7_, yielding significantly enhanced specific capacitance, superior rate capability, exceptional cycling stability, and an optimal balance of high energy and power density, positioning it as a highly viable next-generation electrode material.

## 2. Materials and methods

Manganese nitrate tetrahydrate (Mn(NO_3_)_2_·4H_2_O), ammonium dihydrogen phosphate (ADP) ((NH_4_)H_2_PO_4_), aniline, ammonium persulphate (APS), urea (NH_2_CONH_2_), hydrochloric acid (HCl), acetone (CH_3_COCH_3_), ethanol (C_2_H_6_O), potassium hydroxide (KOH), polyvinylidene fluoride (PVDF), carbon black, *N*-methyl-2-pyrrolidone (NMP), and nickel foam (NF) were all utilized as received (*i.e.*, analytical grade), without requiring additional purification.

### 2.1. Syntheses of MP and PMP

**Fig. 1:**
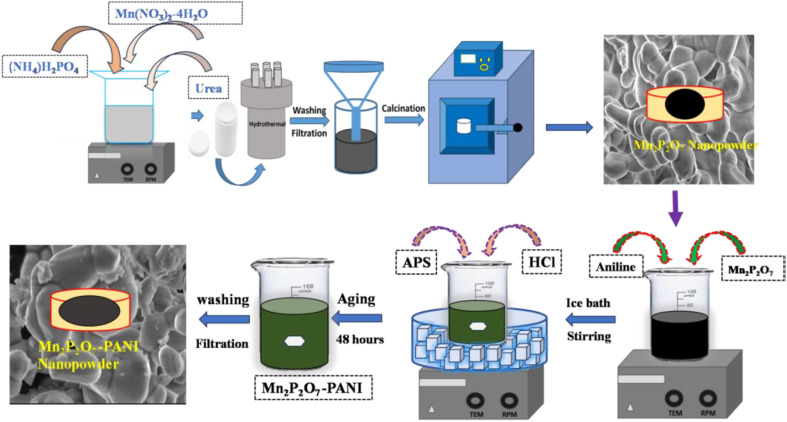
Illustration of the synthesis procedure.

In synthesis format, at the 1st step, MP was synthesized *via* hydrothermal method. In the standard process, a suitable quantity of Mn and P precursors was included in 100 ml of DI water and magnetically stirred. Urea was added to the above solution and continuously stirred for homogeneous formation. The mixture was shifted to a hydrothermal apparatus. In the following procedure, a suitable quantity of aniline monomer was delivered into 100 ml of DI water, which contained a proper amount of the above-collected MP powder, with continuous stirring. Afterwards, the mixed solution was subjected to ultrasonication until a homogeneous mixture was achieved. The whole setup was converted into an ice bath by maintaining 0–50 °C. Meanwhile, HCl (10 ml) was added to the suspension mentioned above at 0–50 °C. After 30 minutes, APS (prepared in 20 ml of DI water) was thoroughly mixed with the suspension, and continuous stirring was performed for 5 hours at 0–50 °C. Afterwards, a dark greenish solution was obtained, suggesting the emeraldine state of PANI, which is known for its more conductive and stable activity. This precipitate was filtered and dried in a hot air oven for an entire night at 650 °C (Fig. 1). The following reaction process is responsible for the formation of MP is as follows. In the 1st stage, manganese nitrate reacted with water molecules while being stirred at room temperature.

In the 2nd stage, Mn^2+^ ions combined with ADP to form a complex compound of MnNH_4_PO_4_·H_2_O. Subsequently, annealing was performed at 8000 °C for 5 hours. The hybrid composite PMP was achieved through electrostatic interaction between PANI and MP. The synthesized MP carries negative charges of P_2_O_7_ on the surface. In contrast, PANI contains amine groups (NH_4_^+^) in its main structure, which facilitate electrostatic solid interaction with the positively charged MP surface during the synthesis process at 0–50 °C.

### 2.2. Instrumentation details

The chemical components of the samples were investigated using Fourier transform infrared spectroscopy (FTIR-Bruker Alpha II) within the range of 500 to 3500 cm^−1^. The samples were analyzed morphologically through field emission scanning electron microscopy (FESEM-JEOL) and high-resolution transmission electron microscopy (HRTEM-JEOL). X-ray photoelectron spectroscopy (XPS-Kratos/Shimadzu Amicus) was performed for elemental analysis.

To create a working electrode, a mixture was made by blending 80%, 10%, and 10% weight of the active material, acetylene black, and PVDF, respectively, in NMP solvent until it formed a uniform slurry. Next, that slurry was coated onto the NF substrate and dried at 75 °C overnight. The NF had approximately 1.6 mg of active material on it. In the first step, the electrochemical tests were performed using a conventional cell system with three electrodes. Active material-coated NF acts as the working electrode. We employed Ag/AgCl and platinum as the reference and counter electrode, respectively. The measurements were performed at room temperature in a 4 M KOH electrolyte solution. In the symmetric two-electrode configuration, the same PMP coated on the NF electrode functions as both cathode and anode components. In a symmetric configuration, 4 M KOH solution was employed as the electrolyte, with a Whatman filter paper serving as the separator. The total active material mass for the balanced device was 3.2 mg. This setup ensures a balanced electrochemical performance and optimizes the overall efficiency of the device. All of the electrochemical tests were executed using the Metrohm-Autolab. The specific capacity, capacitance, power, energy, and coulombic efficiency were estimated using previously published equations.^31–33^

## 3. Results and discussion

### 3.1. Structural and morphological analysis

**Fig. 2:**
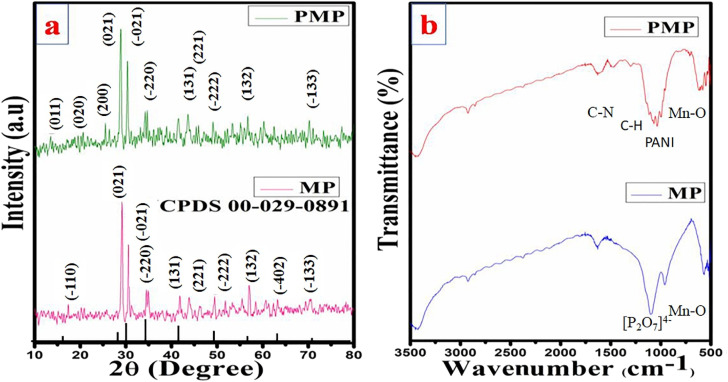
Comparative (a) XRD pattern and (b) FT-IR spectrum.

XRD analysis was applied to investigate the synthesized sample's crystal structure, as shown in Fig. 2(a). The XRD pattern of MP can be matched with the monoclinic phase, and agreed with JCPDS no. 00-029-0891 with space group *C*2/*m*.^31–33^ The presence of distinct diffraction peaks for MP indicates its crystalline nature. PANI exhibited broad peaks in the composite XRD spectra at 2*θ* values of 14.2°, 20.5°, and 25.7°. These peaks can be identified as the (011), (020), and (200) lattice planes, respectively, and indicate the semi-crystalline nature of the PANI chains. The presence of these broad peaks indicates the periodic arrangement of the PANI chains.^34^ During the XRD analysis, it was found that there were no impurity peaks or secondary phases associated with polymerization. However, the peaks of PMP were found to be slightly displaced compared to MP. This could be attributed to the existence of a slight variance in the atomic radii. Based on these findings, it can be concluded that the substitution of PANI in MP does not affect the monoclinic structure.

Before and after the polymerization, the crystallite size was calculated using a previously published report^35^ as 47 nm and 39 nm, respectively. It was apparent that the crystallite size decreasing with the inclusion of PANI over the MP can provide more accessibility of the active space for the intercalation of ions, resulting in easy electrolyte diffusion. FTIR analysis was conducted to obtain more details about the prepared MP and PMP with respect to the chemical components. The IR spectra in Fig. 2(b) show the traces obtained before and after polymerization. The initial form of MP exhibits characteristic bands at 568, 703, 961, and 1110 cm^−1^. The band in 568 cm^−1^ is due to the Mn–O bond (metal–oxygen). The bending modes of PO–P are located in the region around 703 cm^−1^. The bending modes of the symmetric–antisymmetric stretching due to the [P_2_O_7_]^4−^ anion can be identified at 961 and 1110 cm^−1^, respectively. Another band at 1631 cm^−1^ was attributed to the water molecule bending vibrations.^36,37^ These significant bands can also be identified after polymerization with tiny differences in the wavenumber, including other characteristic bands corresponding to PANI. In the context of polymerization, PANI exhibits bands at 757 and 1036 cm^−1^ that are ascribed to the bending C–H vibration of the aromatic ring, and another band at 1281 cm^−1^ that corresponds to the C–N stretching of the secondary aromatic amine. The detected bands at 1482 and 1556 cm^−1^ indicate the stretching vibration of C=C in the benzenoid and quinoid rings, respectively.^38–40^ The FTIR analysis agrees with the XRD results, confirming the preparation of novel composite electrodes of PMP.

**Fig. 3:**
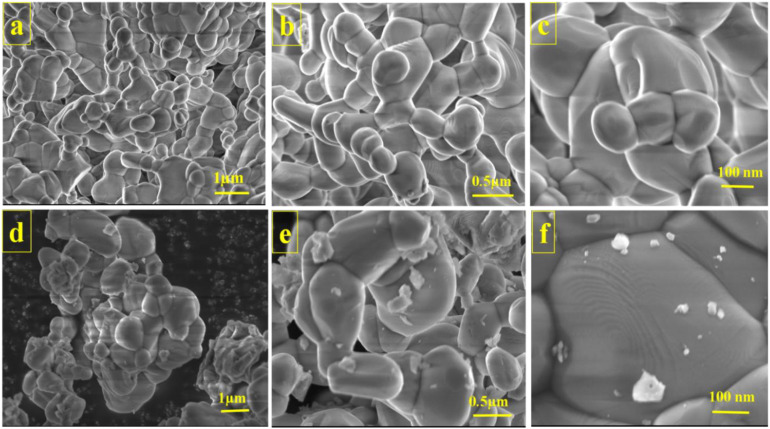
Morphology visualizations of (a–c) MP and (d–f) PMP.

The prepared samples' morphology was confirmed by examining various magnification images from FESEM analysis. Fig. 3(a–f) explains the morphology analysis of the prepared bare MP and its composite PMP. Pure MP forms a nanocluster structure, as can be seen at various magnification rates (Fig. 3a–c). As the formed nanoclusters are interconnected, each one creates an extensive surface area that is suitable for easier diffusion and efficient reactions. The FESEM images of PMP are shown in Fig. 3(d–f). PANI particles grafted on the nanocluster surface can also be found in those images, and are visible at a higher magnification rate (Fig. 3f). Interestingly, after polymerization, MP shows the same structure in terms of its uniform and homogeneous appearance. It is essential to note that incorporating PANI did not significantly change the morphology nature. The MP nanoclusters serve as a host for PANI, creating a large interfacial contact area between the organic PANI and inorganic MP, enabling effective charge transfer and improving the capacitive performance. This hybrid design can handle significant changes in the surface volume, allowing it to effectively reduce the degradation rate of the active material while undergoing cycling. As a result, this enhances the overall durability of the supercapacitors.

**Fig. 4:**
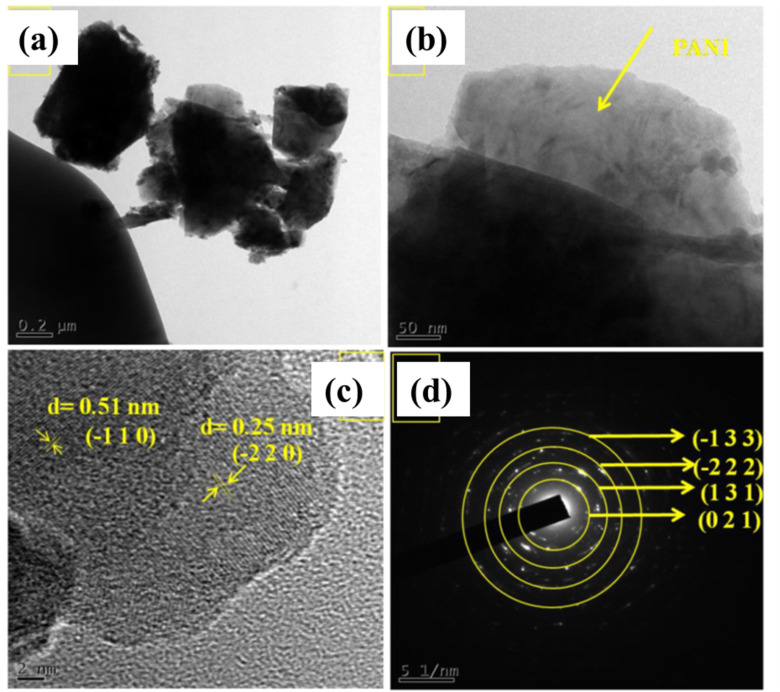
HR-TEM images of PMP (a–c) and SAED pattern of PMP (d).

TEM analysis was carried out to better understand the structural characteristics. These results are displayed in Fig. 4(a–d). Upon conducting a meticulous examination, it has been verified through HRTEM analysis that PANI was safely deposited onto the MP surface, as clearly shown in Fig. 4(a and b). The large surface area and conductive structure are provided by the MP nanoclusters, while the redox activity and charge storage are maximised by PANI. Through this synergy, the supercapacitor performance can be improved. In Fig. 4(c), polymerized MP displays a lattice spacing of 51 and 25 nm, which closely aligns with the (−110) and (−220) planes, respectively, in the monoclinic MP structure. The amorphous nature of PANI resulted in the absence of a lattice structure. In Fig. 4(d), the selected area electron diffraction (SAED) pattern supports the XRD diffractogram, and confirms the monoclinic structure of MP with a highly crystalline nature. This analysis spotlights that PANI is distributed over the MP surface and clearly shows good contact between PANI and MP, confirming that the polymerization technique can effectively create a composite system.

**Fig. 5:**
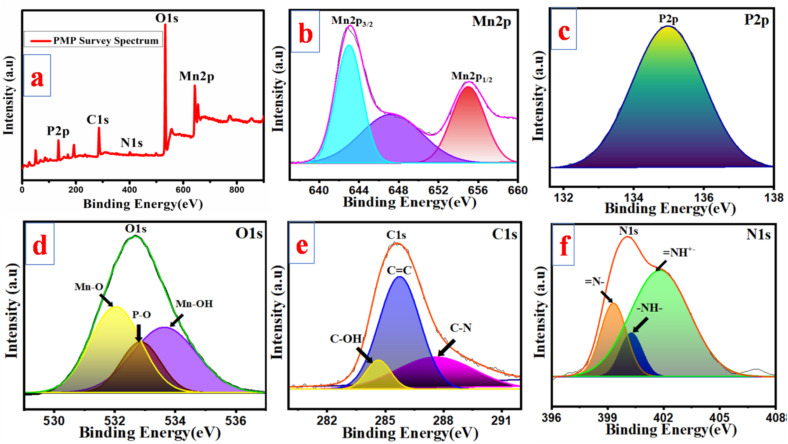
Survey spectrum of PMP (a); spectrum of (b) Mn 2p, (c) P 2p, (d) O 1s, (e) C 1s, and (f) N 1s.

The elemental characterization and chemical composition of the prepared PMP materials were further confirmed by XPS. Fig. 5(a–f) shows the XPS analysis results of PMP. The XPS survey spectrum of PMP (Fig. 5a) reveals peaks for Mn 2p, P 2p, O 1s, C 1s, and N 1s. The peaks at binding energies (BE) of 642.8 and 654.7 eV were linked to Mn 2p_3/2_ and Mn 2p_1/2_ with satellite peaks at 647.5 and 655 eV, respectively (Fig. 5b). This indicates that within the compound, the mixed oxidation states of Mn are +2 and +3. The peak of the P 2p spectra at a BE at 134.9 eV is ascribed to the [P_2_O_7_]^4−^ ion (Fig. 5c).^41,42^ The C 1s spectra (Fig. 5e) exhibit peaks at a BE of 285.4 eV, presenting the hybrid states of carbon such as C–OH, C=C, and C–N. In Fig. 5f, the existence of N 1s is indicated by peaks measured at 399.3, 400.2, and 401.6 eV, which are indicative of imine, amine and nitrogen cationic radical groups, respectively.^43,44^ The presence of PANI over the MP surface is validated by the observation of the N 1s and C 1s spectra.

### 3.2. Electrochemical analysis

#### 3.2.1. Three-electrode configuration

**Fig. 6:**
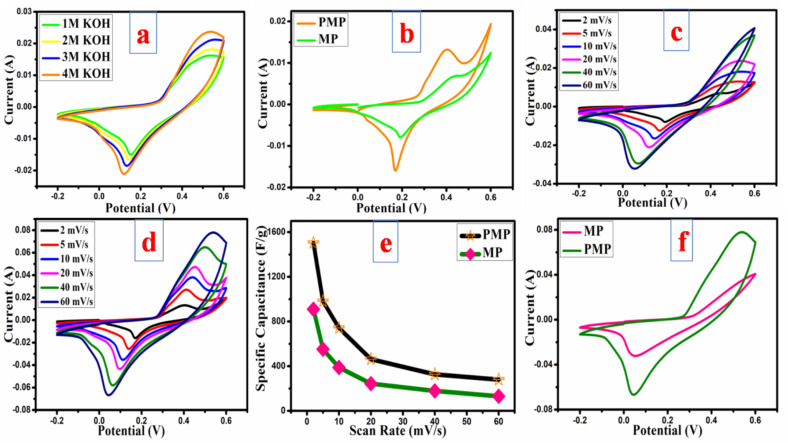
(a) CV performance of MP in different concentrations of KOH electrolyte at 20 mV s^−1^; (b) comparative CV profiles of MP & PMP electrodes at 2 mV s^−1^; CV graphs at various scanning rates for (c) MP, (d) PMP; (e) scan rate *vs. C*_s_; (f) comparative CVs at 60 mV s^−1^.

Initially, the electrochemical behaviour of pristine MP was evaluated by employing CV measurements at a scanning rate of 20 mV s^−1^ with various concentrations of KOH electrolyte (1, 2, 3, 4 M). The MP electrode exhibited enhanced capacitive behaviour in 4 M KOH (Fig. 6a).

Both electrodes (MP and PMP) were electrochemically tested, and their performances were comparatively studied using 4 M KOH electrolyte in the given potential value of −0.2 to 0.6 V at varying scanning rates. At the 1st step, a comparative CV analysis was done at 2 mV s^−1^ (Fig. 6b). Compared to the bare MP electrode, the PMP electrode exhibits prominent redox peaks and improved CV curves, confirming the successful incorporation of PANI. In addition, an enlarged integrated area for the composite electrode indicates the superior electron transport and high energy storage capacity. The individual detailed CV curves of both samples and their corresponding capacitance values at their respective scan rates are summarised in Fig. 6(c–e). In both scenarios, the CV curves demonstrated a shift in anodic and cathodic peaks towards higher and lower potentials, respectively, as presented in Fig. 6(c and d). This shift can be ascribed to the sample's polarization effect and ohmic resistance. The presence of significant redox peaks within the CVs of the PMP electrode, as shown in Fig. 6(d), suggests that there is improved faradaic behaviour because of the addition of PANI. In Fig. 6(e), the capacitance measurements decay with an increment of scan rates, which is possibly due to insufficient time for the electrolyte ions to interact with the active electrode. The CV curves remained similar in shape with a prominent redox peak, even at higher scanning rates compared to the bare material (Fig. 6f), suggesting that the PMP sample possesses exceptional ionic, electronic transport properties and high rate capability.

**Fig. 7:**
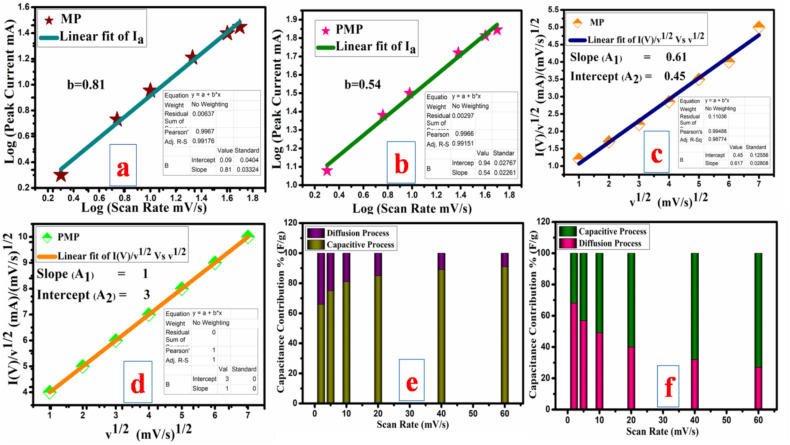
Measured *b* value for both (a) MP and (b) PMP; determined constants (*A*_1_, *A*_2_) for (c) MP and (d) PMP; and specific capacitance contributions at particular scanning rates for (e) MP and (f) PMP.

*I*_p_ is the scanning range (SR)-based peak current (*P*_c_), *ν* is the scanning range (mV s^−1^), and *a* and *b* are constants. The obtained *b* measurements from the slope of the linear fit of log(SR) *vs.* log(*P*_c_) show the dominant charge storage process. If the *b* measurement is approximately 0.5, then the capacitance response is attributed to a diffusion-dominated process. If the value of *b* is around 1, it indicates a capacitive response characterized by surface redox reactions.^45,46^ In this research study, MP exhibits a *b* value of 0.8, which is very close to 1, suggesting that capacitive response-assisted capacitance dominates over the diffusion behaviour. In the case of the PMP electrode, the *b* value is 0.54. This confirms that the capacitance is mainly contributed through diffusion-based redox reactions as shown by the linear fit of *I*(*V*)/*ν*^1/2^*vs. ν*^1/2^ as the slope and intercept, respectively, as represented in Fig. 7(c and d).^47,48^ The particular capacitance contribution corresponding to each scan rate is measured and shown in Fig. 7(e and f). Before polymerization, the MP electrode material delivered 66% capacitive and 34% diffusion contributions. However, after polymerization, the obtained PMP electrode material exhibits 68% diffusion and 32% capacitive response. This noticeable change indicates that a diffusion-assisted redox mechanism plays a more dominant role in the overall charge storage after inclusion of PANI into the MP material. The positively charged nitrogen, quinoid imine, and benzenoid amine in PANI are capable of engaging in redox reactions. Hence, upon including PANI-enriched diffusion behaviour by facilitating additional oxidation states, battery-like behaviour is achieved. Furthermore, as the scan rate increased, the diffusion behaviour decreased, and the main redox peaks slowly disappeared (as can be seen in CV analysis). This might be attributed to insufficient time for the electrolyte ions to interact with the active electrode, resulting in a surface redox reaction. Hence, a capacitive response was developed.

**Fig. 8:**
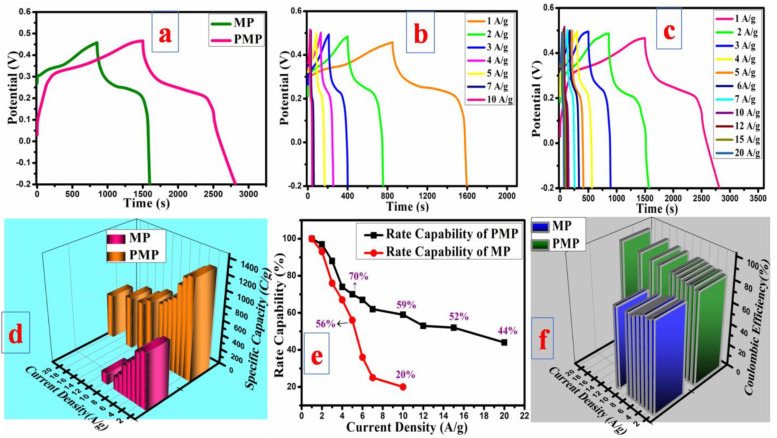
GCD results at 1 A g^−1^ (a), GCD for (b) MP and (c) PMP, (d) 3D graph of current density *vs. Q* bar, (e) rate capabilities for MP and PMP, and (f) coulombic efficiency of MP and PMP.

Fig. 8(a) displays a comparative GCD analysis (1 A g^−1^) for both electrodes. In both, PMP exhibited prolonged discharge time and more extensive discharge region compared to the bare one, which aligns with its excellent performance in CV measurements. The bump within the GCD curves is evidence for the faradaic behaviour, as verified in the CV analysis. Also, the GCD curves support the CV measurements. The *Q* values over all current densities were computed and compared. The MP electrode showed a *Q* value of 750 C g^−1^ at a current density of 1 A g^−1^, whereas PMP delivered a significantly higher *Q* of 1340 C g^−1^. This corresponds to a 1.7-fold increase compared to the bare MP electrode, highlighting the enhanced charge storage capability of the polymerized material (PMP). Surprisingly, the PMP electrode demonstrated a capacity exceeding that of the bare MP electrode at 10 A g^−1^, even when operating at a lower current density of 1 A g^−1^. While moving from low to high current density, there was a slight decay in the capacity due to the constrained time for the electrolyte ions to enter the electrode pores. The rate capabilities and coulombic efficiency were carefully evaluated at all current densities, as provided in Fig. 8(e and f). Interestingly, the PMP electrode showed 59%, 52%, and 44% of the enhanced rate performance at 10, 15, and 20 A g^−1^, respectively, as compared to the bare electrode (Fig. 8e). Notably, PMP demonstrated fabulous coulombic efficiency at higher and lower current densities, suggesting the occurrence of activities within the bulk material.^49,50^ There is a decrease in the coulombic efficiency with rising current density, as observed in Fig. 8(f) for MP. It is ascribed to the limited time available for the electrolyte ions to diffuse into the electrode material at elevated current densities. This led to unwanted reactions like electrolyte collapse, which diminished the overall charge transfer effectiveness.^51,52^ Specifically, after polymerization, the capacity, rate performance, and coulombic efficiency significantly improved due to the good conduction paths, multiple oxidation states, and synergetic effect between PANI and MP, respectively. These studies show that adding an appropriate material to a bare substance significantly improves its effectiveness.

#### 3.2.2. Two electrode system

**Fig. 9:**
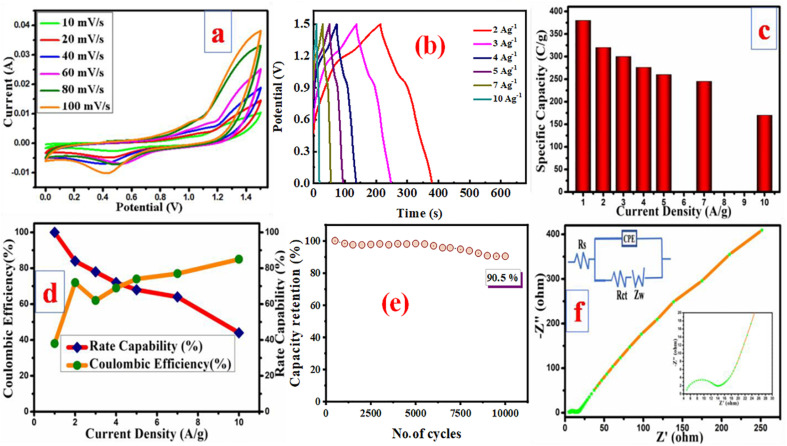
Electrochemical performance of the PMP symmetric device: (a) cyclic voltammograms, (b) charge/discharge profiles, (c) capacity *vs.* current rates, (d) coulombic efficiency and rate performance, (e) stability analysis, and (f) EIS measurements.

The fabrication of a symmetric supercapacitor using the prepared PMP electrode as both positive and negative electrode effectively mitigates the capacitance and voltage window mismatches between the cathode and anode, which are common challenges that are typically encountered in asymmetric supercapacitor devices. The electrochemical performance of the PMP symmetric supercapacitor was evaluated within a voltage range of 0.0 to 1.5 V. This evaluation provides insights into the device's charge storage capabilities and overall efficiency within the specified operational window. Initially, the PMP symmetric device undergoes CV analysis at varying scanning rates within the 1.5 V potential ranges, as illustrated in Fig. 9(a). The consistent shape of the CV profiles across all scan rates signifies that the fabricated symmetric device exhibits exceptional reversibility and robust charge discharge performance, confirming its reliability for energy storage applications. Fig. 9(b) illustrates the charge–discharge characteristics of the PMP symmetric device at varying current densities. The specific capacities derived from GCD curves are 380, 320, 300, 276, 260, 245, and 170 C g^−1^ at corresponding current densities of 1, 2, 3, 4, 5, 7, and 10 A g^−1^, respectively. The device operated at high scan rates and current densities, indicating its potential for practical applications. It retains 44% of its initial capacity at a relatively maximum current density of 10 A g^−1^, as shown in Figure 9(d), showcasing the symmetric device's robust rate performance and suitability for demanding energy storage applications. The system attains a maximum energy density of 79.1 Wh kg^−1^ at a power density of 749.3 W kg^−1^ at a lower current density of 1 A g^−1^. Even at the high current density of 10 A g^−1^, the symmetric device exhibits outstanding rate performance by delivering an energy density of 35.4 Wh kg^−1^, demonstrating its high-performance capabilities under these operating conditions. Furthermore, the fabricated device showed higher coulombic efficiency with the increased current rates, as depicted in Fig. 9(d). The observed increase in the coulombic efficiency with the higher current densities implies that at minimum current densities, the charge storage predominantly occurs within the bulk sample, reflecting the effective utilization of the active material across different operational scenarios. The device durability is further assessed through 10k GCD cycles conducted at 10 A g^−1^, as represented in Fig. 9(e). Even after 10 000 cycles, the device retains 90.5% of its original capacity. This evaluation highlights the outstanding durability and robustness of the device under extended cycling conditions. Additionally, EIS data were analysed using an equivalent circuit model, as presented in Fig. 9(f). In this circuit diagram, *R*_s_, *R*_ct_, and *Z*_W_ represent the solution resistance, charge transfer resistance, and Warburg impedance, respectively, and provide insights into the key resistive and diffusive processes within the system. The measured *R*_s_ and *R*_ct_ values were as low as 4.9 Ω and 7.3 Ω, respectively contributing to the excellent performance.^53–55^ The linear trend observed in the low-frequency region, ascribed to *Z*_W_, indicates the efficient diffusion of the K^+^ ions into the device, reflecting the favourable ion transport characteristics.

**Fig. 10:**
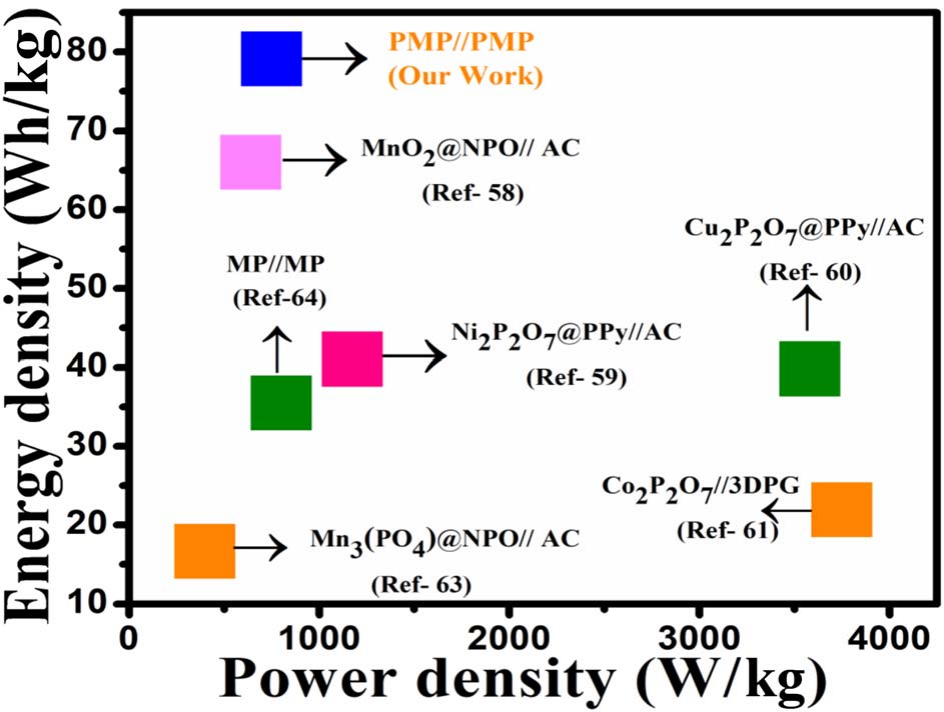
Ragone plot of the PMP symmetric device.

The Ragone plot of the PMP//PMP symmetric device, presented in Fig. 10, is compared with that of the pyrophosphate-based devices to evaluate its performance. Power and energy density are important in real-world applications since they affect the devices' efficiency and operational capacity. Higher energy density signifies extended functional duration, whereas power density represents the device's capability to supply rapid bursts of energy.^56,57^ Both are necessary to ensure desirable device performance. The PMP//PMP symmetric device achieved a high energy density of 79.1 Wh kg^−1^ and power density of 749.3 W kg^−1^, owing to its higher specific capacity and wide potential window. The energy and power density of the PMP//PMP symmetric device are higher than that of other recently outlined pyrophosphate related hybrid devices, including MnO_2_@NPO//AC (66 Wh kg^−1^ at 640 W kg^−1^),^58^ Ni_2_P_2_O_7_@PPy//AC (41 Wh kg^−1^ at 1175 W kg^−1^),^59^ Cu_2_P_2_O_7_@PPy//AC (39.8 Wh kg^−1^ at 3582 W kg^−1^),^60^ Co_2_P_2_O_7_//3DPG (21.9 Wh kg^−1^ at 3750 W kg^−1^),^61^ Mn_3_PO_4_@NPO//AC (16.6 Wh kg^−1^ at 399.3 W kg^−1^),^63^ and MP//MP (35.5 Wh kg^−1^ at 799.9 W kg^−1^),^64^ as shown in Fig. 10.^62^

The *in situ* polymerization at 0–5 °C exploits the electrostatic complementarity: negatively charged [P_2_O_7_]^4−^ groups on Mn_2_P_2_O_7_ (MP) nanoclusters anchor the protonated anilinium ions (NH_4_^+^) during polymerization. This creates a molecular-scale interface that minimizes phase segregation—resolving a key limitation in TMP-conductive polymer composites. The composite induces synergistic redox behavior unattainable by either component alone. MP provides bulk Mn^2+^/Mn^3+^ redox (battery-type, slow diffusion), and PANI enables surface pseudocapacitance (fast kinetics). The explains the 1.78× capacity enhancement (1340 *vs.* 750 C g^−1^) and record energy density (79.1 Wh kg^−1^), outperforming recent hybrids as presented in Table 1. This investigation highlights the importance of the PMP//PMP symmetric device for practical approaches, specifically in improving the energy storage capacity and assuring long-term stable performance.

**Table 1:** Comparison of this work with recently reported hybrids

Material	Energy density (Wh kg^−1^)	Power density (W kg^−1^)	Ref.
PMP (this work)	79.1	749.3	—
MnO_2_@NPO//AC	66	640	58
Ni_2_P_2_O_7_@PPy//AC	41	1175	59
Phosphate/carbide	9.167	1500	65
CeO_2_@PCN//GO	36	753	66
MXene–RBP	55	452	67
CCP//rGO	15.79	1300	68
MgPO_4_–50rGO	21.7	790	69
NiMgPO_4_@CNT//AC	44.5	1030	70
ZnSrPO_4_/N-GQDs	55.68	1500	71
MVO/rGO	38.55	2434.38	72
NCM-NG-2 nanohybrid	31.8	473.4	73
Ni-MOFs/MWCNTs@Ni/Co-LDH	28.9	850	74

## 4. Conclusion

The monoclinic-structured MP and PMP were carefully synthesized through hydrothermal and *in situ* polymerization methods, respectively. The crystal structure and chemical composition were examined utilizing XRD and FTIR, while the morphological features were thoroughly examined by FESEM and HRTEM. Additionally, XPS analysis confirmed the presence of all elements, collectively validating the successful confirmation of PMP. Both electrodes are electrochemically characterized and systematically compared to evaluate their electrochemical features. The engineered PMP electrode material delivers high specific capacity, exceptional cycling stability, and remarkable rate performance in contrast to the performance of pure MP. It was identified that polymerized PMP electrode showed a significantly improved electrochemical performance due to synergy between MP and PANI, its crystalline nature, and their interconnected nanocluster structure. Furthermore, the PMP symmetric device was fabricated and evaluated for its practical applicability. The device showed a maximum energy density of 79.1 Wh kg^−1^ at a power density of 749.3 W kg^−1^, proving its reliability in constructing efficient and sustainable supercapacitors.

## Conflicts of interest

The authors have no conflict of interest that can effect the publication of this article.

## Data Availability

The data will be made available upon request.
